# Transcriptomic analysis of patients with clinical suspicion of maturity-onset diabetes of the young (MODY) with a negative genetic diagnosis

**DOI:** 10.1186/s13023-022-02263-3

**Published:** 2022-03-04

**Authors:** María E. Vázquez-Mosquera, Emiliano González-Vioque, Sofía Barbosa-Gouveia, Diego Bellido-Guerrero, Cristina Tejera-Pérez, Miguel A. Martinez-Olmos, Antía Fernández-Pombo, Luis A. Castaño-González, Roi Chans-Gerpe, María L. Couce

**Affiliations:** 1 Unit of Diagnosis and Treatment of Congenital Metabolic Diseases, Hospital Clínico Universitario de Santiago de Compostela, Santiago de Compostela, Spain; 2 Instituto de Investigación Sanitaria de Santiago (IDIS), Santiago de Compostela, Spain; 3 Centro de Investigación Biomédica en Red de Enfermedades Raras (CIBERER), Madrid, Spain; 4 Universidad de Santiago de Compostela, Santiago de Compostela, Spain; 5 European Reference Network for Hereditary Metabolic Disorders (MetabERN), Padova, Italy; 6 Division of Clinical Biochemistry, Hospital Universitario Puerta de Hierro-Majadahonda, Madrid, Spain; 7 Division of Endocrinology, Complejo Hospitalario Universitario de Ferrol, Ferrol, Spain; 8 Division of Endocrinology and Nutrition, Hospital Clínico Universitario de Santiago de Compostela, Santiago de Compostela, Spain; 9 Centro de Investigación Biomédica en Red de Fisiopatología de la Obesidad y Nutrición (CIBERobn), Madrid, Spain; 10 Endocrinology and Diabetes Research Group, Instituto de Investigación Sanitaria BioCruces, Barakaldo, Spain

**Keywords:** Gene expression, Maturity-onset diabetes of the young (MODY), Nanostring nCounter techonology, Transcriptomic diagnosis

## Abstract

**Background:**

Diagnosis of mature-onset diabetes of the young (MODY), a non-autoimmune monogenic form of diabetes mellitus, is confirmed by genetic testing. However, a positive genetic diagnosis is achieved in only around 50% of patients with clinical characteristics of this disease.

**Results:**

We evaluated the diagnostic utility of transcriptomic analysis in patients with clinical suspicion of MODY but a negative genetic diagnosis. Using Nanostring nCounter technology, we conducted transcriptomic analysis of 19 MODY-associated genes in peripheral blood samples from 19 patients and 8 healthy controls. Normalized gene expression was compared between patients and controls and correlated with each patient’s biochemical and clinical variables. Z-scores were calculated to identify significant changes in gene expression in patients versus controls. Only 7 of the genes analyzed were detected in peripheral blood. *HADH* expression was significantly lower in patients versus controls. Among patients with suspected MODY, *GLIS3* expression was higher in obese versus normal-weight patients, and in patients aged < 25 versus > 25 years at diabetes onset. Significant alteration with respect to controls of any gene was observed in 57.9% of patients.

**Conclusions:**

Although blood does not seem to be a suitable sample for transcriptomic analysis of patients with suspected MODY, in our study, we detected expression alterations in some of the genes studied in almost 58% of patients. That opens the door for future studies that can clarify the molecular cause of the clinic of these patients and thus be able to maintain a more specific follow-up and treatment in each case.

**Supplementary Information:**

The online version contains supplementary material available at 10.1186/s13023-022-02263-3.

## Background

Maturity-onset diabetes of the young (MODY; MIM#606391) is a non-autoimmune monogenic form of diabetes mellitus with characteristic destruction of pancreatic β cells and impaired insulin biosynthesis [1, 2]. MODY classically presents before 25 years of age in individuals with hyperglycemia who do not require insulin, and appears to be inherited in an autosomal dominant manner. In particular, MODY is suspected in normal-weight people who meet these criteria. The absence of these risk factors in type 2 diabetes and the presence of factors specific to type 1 diabetes (autoantibodies against pancreatic cells and low C-peptide levels) can enable differentiation of MODY from other forms of diabetes mellitus (DM) [3]. However, owing to the low incidence of MODY (1–2% of DM cases), these risk factors, including adiposity, are insufficient to conclusively distinguish between these diagnoses, and definitive diagnosis of MODY requires genetic testing [4].

Distinguishing MODY from the two most common types of DM (types 1 and 2) represents an interesting challenge for so-called personalized or precision medicine, as it entails selecting a treatment based on the patient’s etiology. Insulin and metformin administration are the main treatments for DM types 1 and 2, respectively. Hepatocyte nuclear factor-1 alpha (HNF1A)-MODY (MIM#142410) and, in some cases, HNF4A-MODY (MIM#600281) are treated with low-dose sulfonylureas, a low-cost oral treatment [5, 6]. Glucokinase (GCK)-MODY (MIM#138079) usually results in increases in basal blood glucose levels that do not give rise to the common sequelae of DM, and generally does not require pharmacological treatment [7]. These are the three most common manifestations of MODY, and can be extrapolated to other variants of the disease, in which up to 19 genes can be affected [8]. Personalized management of each type of MODY results in better patient care and the avoidance of invasive therapies such as insulin in favor of more effective and economical treatments. It is thus possible to better determine the patient’s prognosis and carry out family screening to avoid future erroneous diagnoses [9].

The exome sequencing is the most widely used tool to study variants in the genes associated with different MODY subtypes: *ABCC8*, *APPL1*, *BLK*, *CEL*, *GCK*, *HNF1A*, *HNF1B*, *HNF4A, INS*, *KCNJ11*, *KLF11*, *NEUROD1*, *PAX4*, and *PDX1* [10]. Although genetic testing is a fundamental tool for the differential diagnosis of this type of diabetes, it may be insufficient. Some studies indicate a 50% success rate in genetic diagnosis in probands with suspected MODY [11], although this may be an overestimate: a UK study reported a positive genetic diagnosis in only 27% of 2072 individuals with the MODY phenotype [12]. This suggests that certain forms of MODY are caused by variants that affect gene expression and are not detectable by exome-directed analysis that therefore cannot be diagnosed using standard methods. It should be noted large portions of the human genome are involved in regulating transcription [12, 13], and that 5′ and 3′ untranslated regulatory regions, non-coding RNAs (ncRNAs), and transcriptional enhancers, silencers, and isolators can exert significant effects on gene expression patterns that can only be revealed by transcriptomics [14]. Recent studies have demonstrated an increase of up to 33% in the rate of diagnosis of rare diseases using transcriptomics [15], which, combined with genomics, constitutes an essential tool for the molecular diagnosis of many diseases. Together, these techniques can reveal genotype–phenotype correlations, uncover gene expression profiles associated with a given genetic condition, and enable immediate evaluation of the effects of genomic variants on gene expression [15, 16].

The most commonly used technology for transcriptomic analysis is RNA-seq, which reveals the presence and quantity of RNA through massive sequencing. Nanostring nCounter technology is an alternative to RNA-seq [17, 18] that is widely used to study gene expression, especially in cancer [19–21]. This multiplex nucleic acid hybridization technology is highly reproducible and less dependent on RNA quantity and quality [17–22].

In this study, we evaluated the utility of transcriptomic analysis with Nanostring nCounter as a complementary tool to diagnose patients with a MODY phenotype for whom standard genetic testing failed to establish a diagnosis. Moreover, we conducted this analysis using peripheral blood samples, which can be acquired quickly, easily, and noninvasively, allowing easy incorporation of this methodology into routine clinical testing.

## Results

### Clinical and biochemical characteristics of the patients

Table 1 shows the data collected for each of the 19 participating patients (11 men and 8 women; mean age at enrolment, 47.2 ± 15.3 years). The mean age of onset of diabetes was 30.1 ± 13.8 years, and 78.5% had a family history of DM. Fasting glycemic status was normal in 21.1% of patients, and 21% had abnormal C-peptide values. 36.8% presented obesity, and 26.32% were overweight. Eighteen of the 19 participants were receiving antidiabetic treatment, the most common of which was insulin treatment. Five of these 18 participants were being treated with combination therapy consisting of 2 or more drugs. Assuming target HbA1c values < 7% for individuals receiving hypoglycemic treatment [23], 63.2% of patients in our cohort had poor glycemic control. The 8 controls (5 men and 3 women), in the same age range as the patient cohort, did not have any associated metabolic disease or alteration of the biochemical parameters collected for the study.

**Table 1:** Clinical and biochemical characteristics of the patients

Case	Age (years)	Sex	Age at onset (years)	Family history of DM (n)	BMI (Kg/m^2^)	Biochemical parameters			Secondary complications	Antidiabetic therapy				
						HbA1c (%)	Fasting glucose (mg/dl)	C-peptide (ng/ml)		Insulin	Metformin	DDP4i	SGLT2i	GLP-1 RAs
M1	61	M	48	7	31.8	7.2	110	1.1	No	Yes	No	No	Yes	No
M2	85	M	49	4	19.6	7	126	0.8	Yes	No	Yes	No	No	No
M3	47	M	27	5	25.1	6.9	128	0.5	Yes	Yes	Yes	No	Yes	No
M4	78	F	65	9	24.3	7.6	90	0.3	No	Yes	No	No	No	No
M5	34	F	25	2	20.8	7.5	122	1.8	No	No	No	Yes	No	No
M6	35	F	23	5	30.6	8.2	113	1.6	No	Yes	No	No	No	No
M7	53	M	18	2	25.6	8.2	142	1.3	Yes	Yes	Yes	Yes	No	No
M8	46	M	26	1	19.8	6	125	0.3	No	Yes	No	No	No	No
M9	35	M	14	–	27.5	8	191	0.7	No	Yes	No	No	No	No
M10	32	F	20	2	40.2	9.8	138	0.4	No	Yes	Yes	No	Yes	No
M11	55	M	27	6	27	6.9	145	–	No	No	Yes	No	No	No
M12	48	F	25	2	30.4	5.9	102	3	No	Yes	No	No	No	No
M13	48	M	46	3	24.8	6.8	159	0.8	No	No	Yes	No	No	No
M14	24	M	17	4	23.6	7.6	127	1.4	No	No	Yes	No	No	No
M15	42	F	11	3	38.7	6.2	90	0.57	Yes	No	No	No	No	Yes
M16	57	M	35	2	25.5	7.9	167	1.2	No	Yes	No	No	No	No
M17	38	F	35	–	29.1	7.3	124	0.5	No	Yes	No	No	No	No
M18	41	F	25	–	27.2	8.4	143	–	No	No	No	No	Yes	No
M19	38	M	35	2	22	6.4	124	1.4	No	No	No	No	No	No
C1	40	M	–	0	23.1	–	93	–	No	No	No	No	No	No
C2	35	F	–	0	20.1	–	92	–	No	No	No	No	No	No
C3	30	F	–	0	22.4	–	101	–	No	No	No	No	No	No
C4	58	M	–	1	19.4	–	96	–	No	No	No	No	No	No
C5	46	M	–	0	23.7	–	89	–	No	No	No	No	No	No
C6	39	M	–	1	24.6	–	88	–	No	No	No	No	No	No
C7	61	F	–	1	20.8	–	92	–	No	No	No	No	No	No
C8	47	M	–	0	22.9	–	90	–	No	No	No	No	No	No

*BMI* body mass index, *DM* diabetes mellitus, *HbA1c* glycosylated hemoglobin A1c, *DDP4i* dipeptidyl peptidase 4 inhibitors, *M* male, *F* female, *n* number, *SGLT2i* sodium glucose cotransporter 2 inhibitors, *GLP-1 RAs* glucagon-like peptide type 1 receptor agonist

### Gene expression results

The results obtained using the nCounter Nanostring platform were standardized using negative controls (background threshold) and a positive control that allowed for normalization of differences due to variations in hybridization efficiency. Next, counts were normalized against RNA content in each case, using housekeeping genes selected by the GeNorm algorithm. After normalizing the counts obtained for each transcript we found that only 7 of the 19 genes included in the panel were detectable in blood: *APPL1*, *BLK*, *GLIS3*, *HADH*, *IER3IP1*, *PLAGL1*, and *UCP2*.

**Fig. 1:**
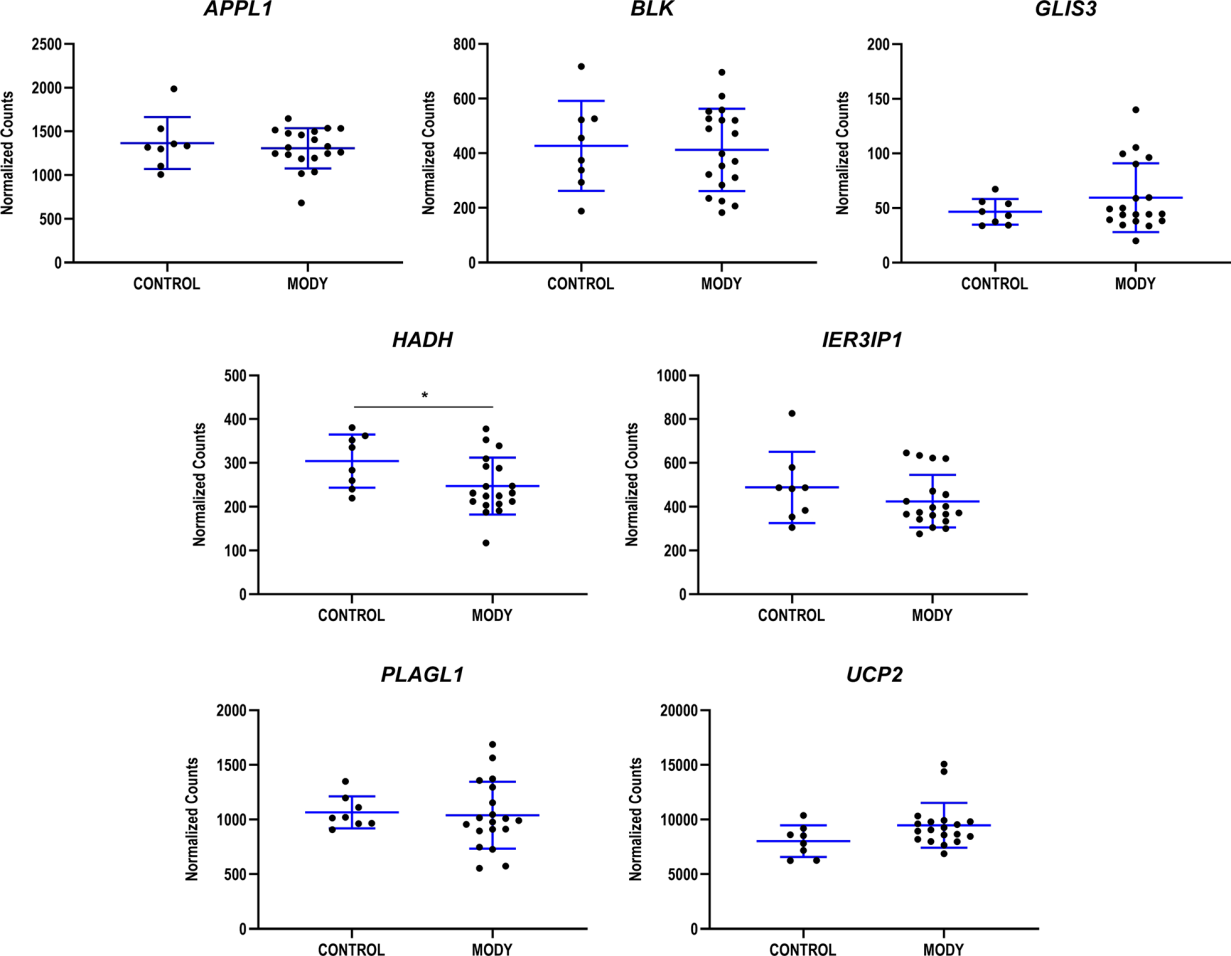
Gene expression, expressed as normalized counts, for each of the genes analyzed. Data are expressed as the mean ± standard deviation for each group (controls and patients with suspected MODY). *p < 0.05

Before analyzing individual gene expression profiles, we compared normalized expression levels between controls and patients to detect global differences in gene expression between the 2 groups. *HADH* expression was significantly (*p* < 0.044) lower in patients with suspected MODY versus controls (Fig. 1). Expression levels of the remaining genes studied were comparable in patients and controls.

**Fig. 2:**
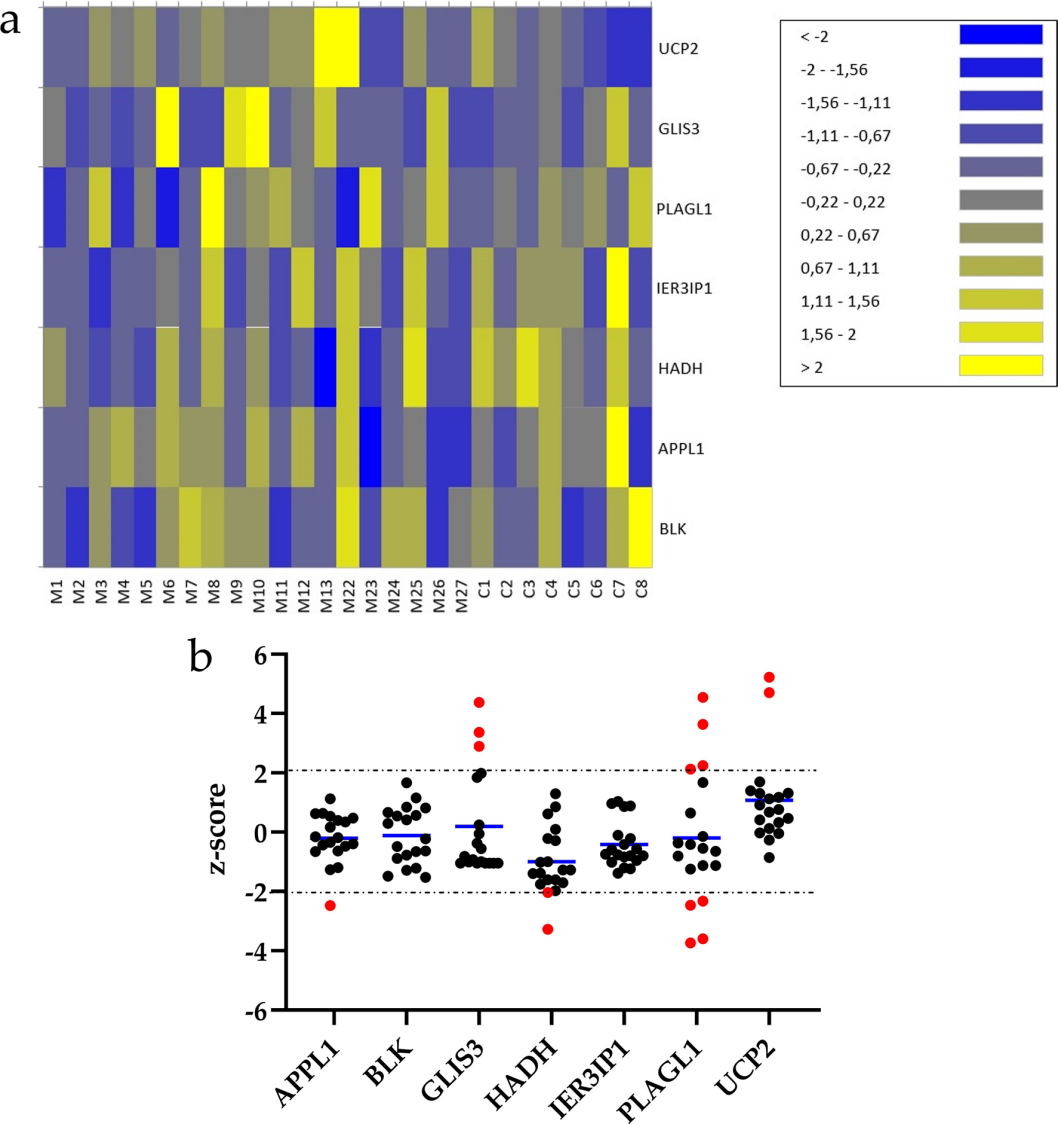
Gene expression z-score, calculated relative to the mean count obtained in the control group. **a** Heatmap depicting color-coded z-scores for each gene analyzed in each patient. **b** Graph depicts the mean z-score for each gene. Red values indicate genes for which marked alterations in expression were observed (z-score ≥ 2 or ≤  − 2)

To analyze the gene expression patterns individually, for each gene analyzed in each patient we calculated a z-score based on the mean count obtained in the control group. A z-score ≥ 2 indicates overexpression of the gene, while a z-score ≤  − 2 indicates low gene expression. Changes in gene expression were observed in 11 of the 19 patients in our cohort, as shown in both the heatmap (Fig. 2a) and the distribution graph (Fig. 2b). No alterations in the expression of *BLK* or *IER3IP1* were observed in any patients. For *APPL1,* the z-score limit (− 2.48) was exceeded in 1 patient (M15). *HADH* expression differed significantly between controls and patients, and the z-score obtained for patients M15 (− 3.27) and M23 (− 2.03) was well below the limit. The gene for which the greatest change in expression was observed in the MODY group was *PLAGL1,* for which we observed two distinct expression profiles: a decrease in expression in patients M1 (− 2.46), M4 (− 2.32), M6 (− 3.59), and M14 (− 3.73); and an increase in patients M8 (4.54), M15 (3.63), M18 (2.12), and M3 (2.24). *GLIS3* overexpression was observed in 3 patients: M10 (4.38), M6 (3.36), and M9 (2.89). *UCP2* was overexpressed in 2 patients: M13 (5.22) and M14 (4.7). In 3 patients with suspected MODY (M6, M14, and M15) we observed altered expression of more than 1 gene.

### Correlations between patient genetic, clinical, and biochemical characteristics

**Fig. 3:**
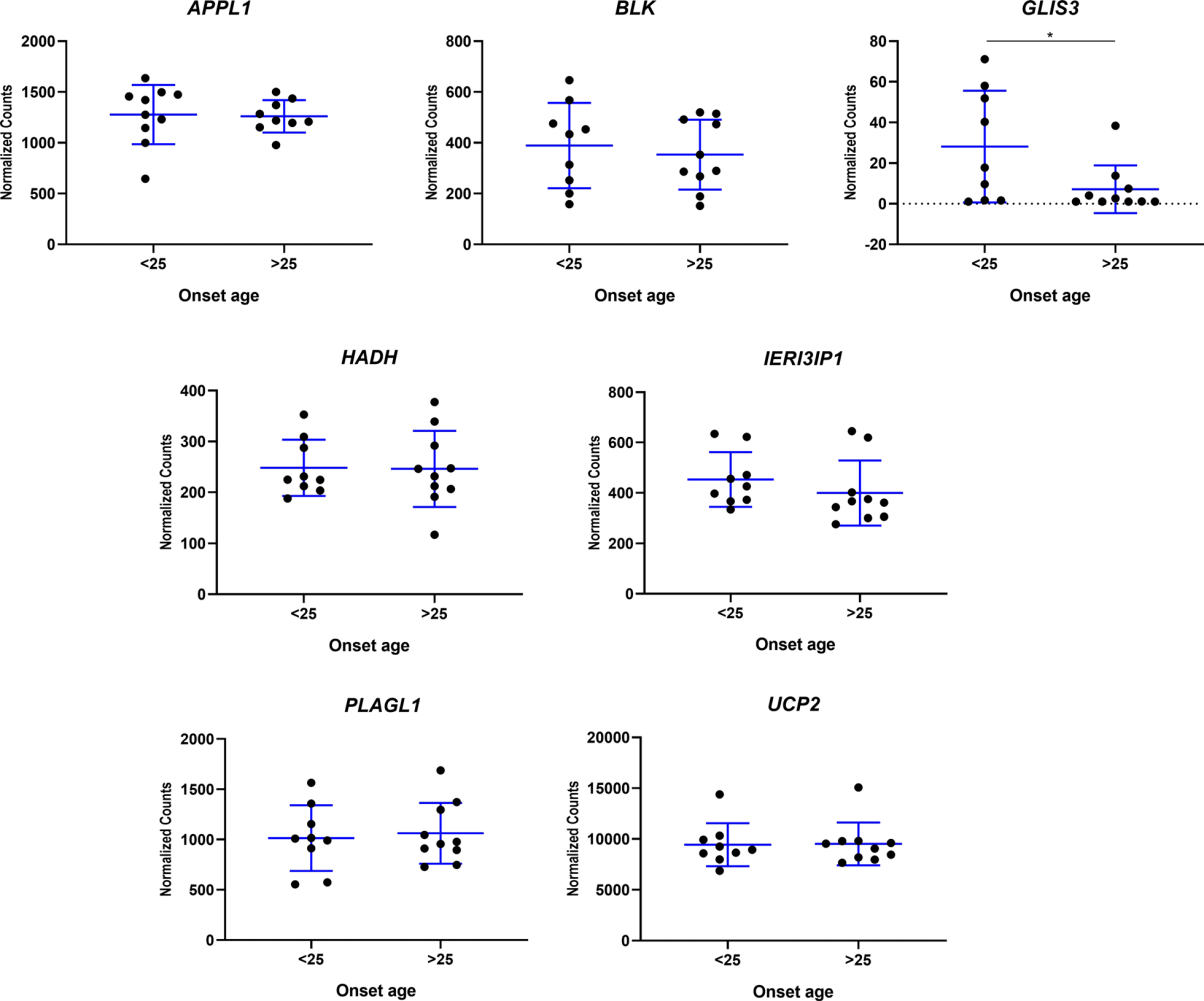
Gene expression, expressed as normalized counts, for each of the genes analyzed in the study in patients with suspected MODY. Results are stratified based on age at onset (< 25 years vs. > 25 years). Data are expressed as the mean ± standard deviation for each group. *p < 0.05

We observed no global correlation between expression levels of any of the genes analyzed and fasting glucose, HbA1c, or C-peptide concentrations. However, stratification according to age at onset, applying a cut-off value of 25 years, revealed that *GLIS3* expression was significantly higher (*p* = 0.041) in patients with an age at onset ≤ 25 years versus those with an age at onset > 25 years (Fig. 3).

**Fig. 4:**
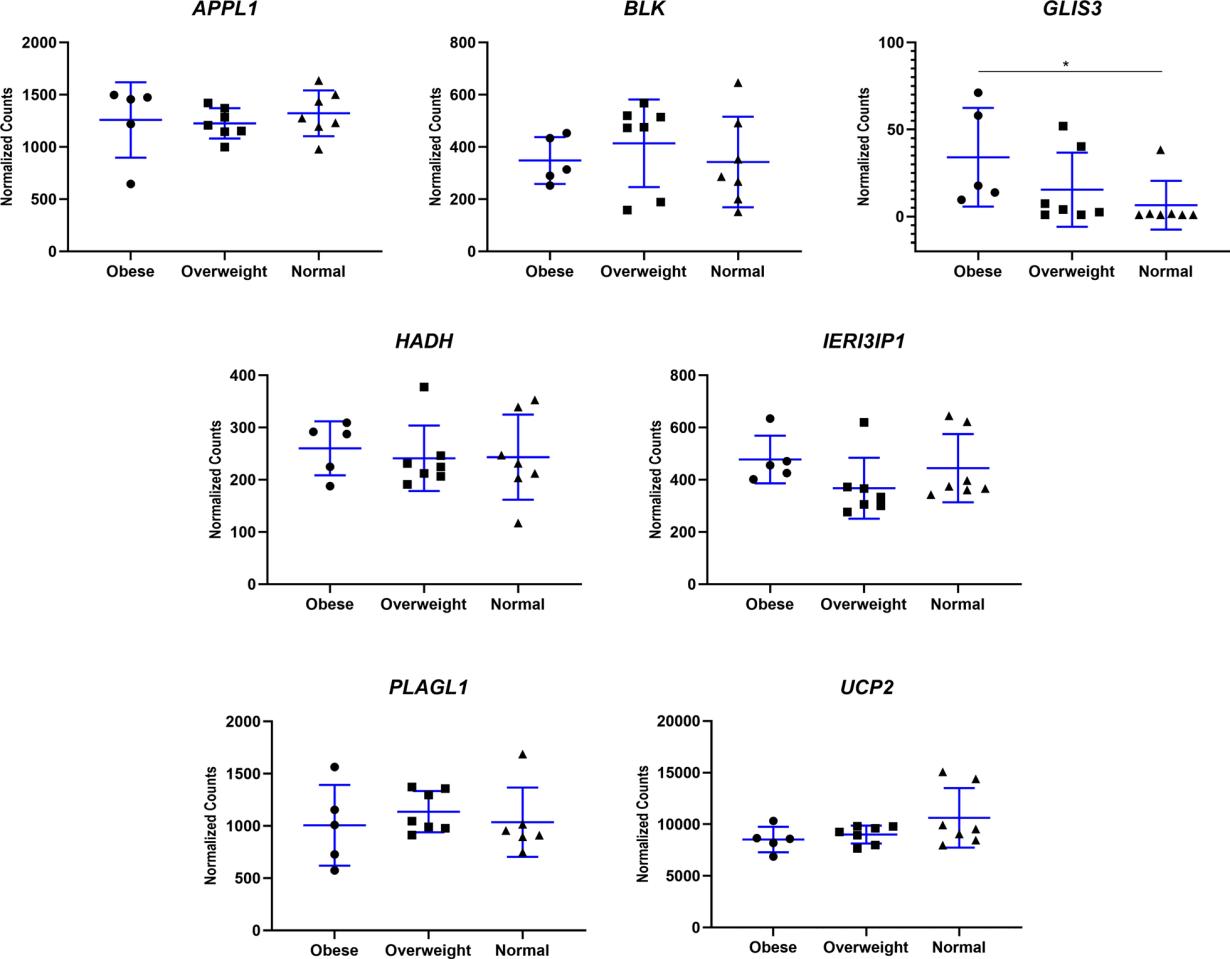
Gene expression, expressed as normalized counts, for each of the genes analyzed in the study. Graphs compare patients with suspected MODY, stratified according to body mass index (BMI): BMI ≥ 30 kg/m^2^ (Obese); BMI = 25–29.99 kg/m^2^ (overweight); BMI < 24.99 kg/m^2^ (normal). Data are expressed as the mean ± standard deviation for each group. *p < 0.05

In addition, comparison of obese patients with patients with a normal BMI revealed significantly higher *GLIS3* expression (*p* = 0.017) in the former group (Fig. 4).

## Discussion

Classification of MODY can often be difficult due to the presence of clinical features that are also found in DM types 1 or 2. Increasing the precision of MODY diagnosis could help avoid unnecessary expensive and invasive tratments and improve the quality of life of these patients’ [5, 7]. Currently, exome sequencing is the most widely used tool to study variants in the genes associated with different MODY subtypes [10]. However, a substantial proportion of patients with clinically suspected MODY remains undiagnosed due to negative genetic diagnoses [11].

We sought to evaluate the efficacy of a new tool that could increase the rate of MODY diagnosis and thereby enable selection of optimal personalized treatments [24]. We investigated whether transcriptomics analysis of patients lacking a positive genetic diagnosis could increase the diagnostic efficacy of massive exome sequencing.

Transcriptomics is widely used as a support tool to validate candidate genes of diagnostic utility in certain diseases. By enabling verification of alterations in gene expression profiles in a given disease, compared either with other diseases or with healthy controls, transcriptomics perfectly complements genetic testing for the diagnosis of hereditary diseases [15, 16, 25]. Non-coding DNA harbors various regulatory sequences that control gene transcription [26]. The complex process of gene regulation is mediated at several levels, including regulation in cis by promoters, enhancers, and repressors and regulation in trans by transcription factors or microRNAs [27]. Therefore, an orthogonal transcriptomic-genomic approach can reveal genotype–phenotype correlations, uncover gene expression profiles associated with the genetic condition of interest, and allow immediate evaluation of the effects of genomic variants on gene expression [28]. The ultimate goal of incorporating transcriptomics into genetic testing is to increase the diagnostic rate. Several studies have achieved this goal using massive RNA sequencing, successfully identifying the genetic cause of the disease in at least 10% of cases in which exome sequencing results were negative [15, 16, 29]. The main events that can be detected with transcriptomics are splicing, allele imbalance, and extreme expression values [30, 31]. Given that most current exome analysis technologies can detect splicing events and that allele imbalance is largely irrelevant in MODY (since most of the genes involved are inherited in an autosomal dominant manner), the greatest utility of transcriptomics in this context is to detect extreme expression of specific genes [17, 29, 31]. This is one of the reasons why we selected the Nanostring nCounter platform: it offers a high degree of technical reproducibility, has lower sample quantity and quality requirements and is less expensive than RNA-seq, and can be easily incorporated into routine clinical testing [32]. For this, we chose blood as the sample for this assessment, as it is the least invasive, comfortable, and easy to obtain sample collection in the case of wanting to establish this technique in the routine of genetic diagnosis.

Expression of 12 of the 19 genes included in our analysis was undetectable in blood samples, and therefore we were unable to characterize the gene expression profile associated with the main types of MODY. This is one of the significant limitations of transcriptomics as a routine or complementary tool for genetic diagnosis in MODY disease. Ideally, such an analysis would be performed with a sample that can be easily obtained from patients using a minimally invasive procedure. However, because gene expression is tissue-dependent, the use of whole blood is conditioned by the expression of genes of interest in this tissue. Some studies have reported an increase the diagnostic rate for rare diseases after incorporating transcriptomics into genetic diagnostic testing [15, 29]. However, this increase was lower when blood or fibroblast samples were used, and higher when muscle samples were used [15]. Despite their accessibility, blood samples may provide the least information of all sample types in the context of genetic analysis.

In our study, in cases in which genomics does not confirm suspected MODY, transcriptomics will not increase the diagnostic rate if blood samples are used. However, it is striking that most of genes that were detected in blood samples (*APPL1, HADH, BLK, IER3IP1, PLAGL1, GLIS3,* and *UCP2*) showed changes in expression in 57.9% of patients. Taking into account that for most of the genes studied, it is the defects in their expression that are associated with diabetes; we look at those cases in which we detect aberrant gene expression. *APPL1* is a leucine 1 zipper motif and a positive regulator of insulin-stimulated Akt phosphorylation and glucose uptake in adipose tissue, muscle, and liver [33, 34]. Pathogenic variants in *APPL1* are associated with MODY type 14 (MIM#616511) [35]. Patient M15 in our cohort showed decreased *APPL1* expression relative to controls, and markedly lower expression than the other patients in the cohort. Given the function of this gene and its implication in MODY type 14, *APPL1* is a potential cause of diabetes in this patient. Intronic variants in this gene that could not be detected in genomic analyses have been described in MODY patients [36].

*HADH* (L-3-hydroxyacyl-coenzyme dehydrogenase) catalyzes the oxidation of straight-chain 3-HAhydroxyacyl-CoAs as part of the beta-oxidation pathway, with the highest enzymatic activity with medium-chain-length fatty acids. Pathogenic variants in this gene are associated with familial hyperinsulinism (MIM#609975) [37]. We observed that, except for four, the MODY subjects have negative z-score values in our cohort. However, only two of them exceed the cut-off point established to consider a significantly low expression of this gene. This was expected since for this gene, in the initial comparison between groups, we already detected that HADH had significantly lower expression in MODY than in controls. Although an association between diabetes and common specific variants in this gene has not been demonstrated [38], altered *HADH* expression has been described in diabetes [39]. Wich suggests a possible role for this enzyme in the disease and coincides with the difference in expression concerning controls found in the MODY patients in our study.

The Glis family zinc protein 3 (*GLIS3*) functions as an activator and repressor of transcription. Loss of *GLIS3* results in a drastic reduction in insulin expression, leading to hyperglycemia that subsequently induces beta-cell apoptosis and culminates in severe fulminant diabetes. [40]. In this study, we do not have any case with aberrant expression of *GLIS3* that could guide the diagnosis in this sense. However, *GLIS3* expression was significantly higher in obese versus normal-weight patients, and in patients aged < 25 versus > 25 years at diabetes onset. GLIS3 expression is required for compensatory pancreatic cell proliferation and mass expansion in response to insulin resistance [41]. This fact may explain the significant increase in expression in patients with an earlier onset in which pancreatic function may be more compromised and in obese patients in whom insulin resistance may be more evident. Given that *GLIS3* is a crucial transcription factor in beta cells and is essential for normal beta cell function in adulthood [40], *GLIS3* may play an important role in a subpopulation of diabetic patients with a normal BMI and later-onset illness.

*PLAGL1* encodes a C2H2 zinc finger protein that functions as a suppressor of cell growth and is a strong candidate for transient neonatal diabetes mellitus (TNDM1; MIM#601410) [42]. Patients with TNDM1 develop hyperglycemia during the first week of life, although diabetes resolves spontaneously during the first 18 months [43]. A considerable proportion of patients with TNDM1 acquire permanent diabetes during adolescence or young adulthood, possibly as a consequence of residual pancreatic dysfunction [44]. The etiology of this disease includes alterations in the imprinted 6q24 locus that lead to overexpression of the imprinted genes *PLAGL1* and *HYMAI.* In our cohort of patients, we found two clear trends in the expression of this gene, with four patients in whom the expression is markedly lower and four in whom there is an evident overexpression of PLAGL1. We did not find any association of these tendencies with the patients' clinical variables that could explain these two very marked profiles. Therefore, we pay special attention to the four patients whose PLAGL1 is overexpressed (particularly evident in patients M8 and M15) since this trend would explain a possible association with MODY and not the opposite. For none of these cases is there evidence that they have had a diagnosis of TNDM1 in their childhood. We cannot rule out this possibility as some patients with TNDM1 do not develop neonatal diabetes due to incomplete penetrance [43, 45].

This is the first study to employing transcriptomics analysis in patients with suspected MODY with a negative genetic diagnosis. Our findings indicate that blood samples are not valid for this type of analysis, as expression of the vast majority of genes associated with MODY is not detectable in blood. We were therefore unable to assess the utility of this tool variation as a means of increasing the diagnostic rate. Nonetheless, in our cohort Nanostring nCounter technology identified extreme expression of genes of interest in 31.6% of patients analyzed. These findings can serve as a useful starting point to identify the molecular targets causing disease in these patients.

The main limitation of our study was the use of peripheral blood samples, in which we were unable to detect expression of 12 of the 19 MODY-associated genes. Although we did detect extreme expression of several genes in patients versus controls, further analyses in a larger study population will be necessary to clarify the diagnostic utility of transcriptomics in this context. Nanostring nCounter technology was selected based on its simplicity, sensitivity, and easy implementation into routine testing protocols. However, this technique is only capable of detecting extreme expression of genes of interest, and not splicing events nor allele imbalance.

## Conclusions

Peripheral blood transcriptomic analysis is not an excellent tool to increase the diagnosis rate in patients with suspected MODY with a negative genomic study because it cannot detect the expression of all associated genes in this tissue. Analysis using Nanostring nCounter technology of genes for which extreme expression was observed identified candidate causative genes in more than 30% of patients.

## Methods

### Study design and population

This cross-sectional observational multicenter study was carried out using data obtained from 19 patients with suspected MODY. Participants were recruited at the 3 participating Spanish hospitals. The inclusion criteria for omics analysis were clinically suspected MODY and a negative genomic diagnosis to date. Suspicion of MODY included the absence of islet autoantibodies (glutamate decarboxylase [GAD] and islet antigen-2 [IA-2] antibodies), the preservation of endogenous insulin secretion and at least one of the following: (1) early age of diagnosis (≤ 35 years); (2) a positive, multigenerational family history of diabetes following an autosomal dominant inheritance pattern [46]. The study period ran from January 1, 2020, to February 28, 2021. The control group consisted of 8 healthy individuals in same age range as the patient cohort, without metabolic disease or alterations in the biochemical parameters measured in the study. The study was approved by the Santiago-Lugo Research Ethics Committee (code: 2019/370), and patients and controls provided written informed consent.

The following variables were recorded for each patient: family history of DM; sex; age at recruitment and at clinical onset; anthropometric characteristics including weight, height, and body mass index (BMI); DM-related blood biochemistry parameters (fasting glucose, glycosylated hemoglobin (HbA1c), and C-peptide); and pharmacological treatment. The presence or absence of secondary complications, both microvascular (retinopathy, nephropathy, neuropathy) and macrovascular (ischemic heart disease, stroke, peripheral arthropathy), was also recorded, in addition to gene expression data obtained using the Nanostring nCounter platform. Clinical data such as the family history of DM, age at clinical onset, pharmacological treatment, and the presence or absence of secondary complications were collected from the patient’s electronic medical records.

### Anthropometric and analytical measurements

Patients were weighed in the morning before eating. Qualified personnel measured standing height using a wall-mounted stadiometer and body weight using a digital scales. Nutritional status was assessed by calculating BMI using the following formula: BMI (m^2^) = weight (kg)/height^2^. Patients were classified according to World Health Organization (WHO) criteria as underweight (BMI < 18.5), normal weight (BMI 18.5–24.99), overweight (BMI 25–29.99), or obese (BMI ≥ 30) [47].

C-peptide levels were measured using the ADVIA^®^ Centaur XP immunoassay system (Siemens). The ADVIA^®^ Chemistry XPT was used to measure glucose levels, and HbA1c was measured using the D-100 TM hemoglobin testing system (Bio-Rad). Reference ranges for the biochemical markers were as follows: glucose 74–105 mg/dL, Hb1Ac < 5.7%, and C-peptide 0.81– 2.85 ng/mL.

### Samples and RNA extraction

Blood samples were received from each of the participating centers within 48 h of extraction. These samples were collected in PAXgene^®^ tubes (Qiagen) to guarantee RNA stability. RNA from each sample was obtained using the PAXgene^®^ Blood RNA Kit (Qiagen), following the manufacturer’s instructions. To assess both the quantity and quality of RNA, the samples were analyzed with the Agilent 2200 TapeStation system, ensuring a RIN (RNA integrity number) > 8 in each case.

### Quantification of gene expression

The nCounter^®^ (Nanostring) platform was used to quantify the number of copies of candidate genes for diagnosis of MODY. This assay consists of hybridization, without the need for amplification, of specific probes of each transcript: one with biotin at the 3′ end that allows detection and another reporter probe containing a unique color code at the 5′ end that identifies the gene. After hybridization and removal of excess probes, the probe-target complexes are bound, immobilized, and aligned on the nCounter cartridge [18]. A specific panel was designed to detect the 19 genes included in the panel used for clinical exome sequencing (CES): *ABCC8*, *APPL1, BLK*, *CEL*, *GCK*, *GLIS3*, *HADH*, *HNF1A*, *HNF1B*, *HNF4A*, *IER3IP1*, *INS*, *KCNJ11*, *KLF11*, *NEUROD1*, *PAX4*, *PDX1*, *PLAGL1,* and *UCP2*. The transcripts quantified and the specific probe and its position are detailed in Additional file 1: Table S1. A series of candidate housekeeping genes was also included in order to normalize gene expression data (Additional file 2: Table S2): *ABCF1*, *ALAS1*, *EEF1G*, *G6PD*, *GAPDH*, *GUSB*, *HPRT1*, and *TBP*. After confirming that all technical parameters fulfilled Nanostring requirements, technical normalization was carried out using the panel negative controls, establishing a background threshold (mean + 2 standard deviations); and using the positive controls to establish a normalization factor to correct for differences due to variations in hybridization efficiency. Finally, we normalized our data based on RNA content, using the GeNorm algorithm to select the optimal number of the most stable reference genes. Ultimately, *ABCF1, ALAS1, GAPDH, GUSB, TBP* were chosen as reference genes.

Z-scores were calculated to identify significant changes in gene expression in patients versus controls. For this, the following formula was used: z-score = (x − µ)/σ, where “x” is the sample normalized counts; “µ”, the mean of normalized counts of controls; and “σ” the standard deviation of normalized counts in the control population.

### Statistical analysis

Appropriate statistical analyses were performed using GraphPad Prism software (version 6). Differences between groups means were analyzed using an unpaired t-test or, in cases in which the data did not follow a normal distribution, a non-parametric Mann–Whitney U-test.

Pearson’s correlation coefficient was used to evaluate correlations between gene expression data and biochemical and anthropometric parameters, except in cases in which normal distribution could not be assumed, in which case Spearman’s non-parametric correlation was used.

Data are presented as the mean ± SD. A *p* value < 0.05 was considered significant.

## Supplementary Information

**Additional file 1: Table S1.** Presents information on the genes analyzed using the Nanostring nCounter platform, including the corresponding reference sequence and target position.

**Additional file 2: Table S2** Presents housekeeping genes included in the panel for nCounter-Nanostring analysis.

## Data Availability

The datasets used and/or analysed during the current study are available from the corresponding author on reasonable request.
