# Supplementary material for: Transcriptomic analysis of patients with clinical suspicion of maturity-onset diabetes of the young (MODY) with a negative genetic diagnosis
Source: Orphanet J Rare Dis. 2022 Mar 4;17:105. doi: 10.1186/s13023-022-02263-3 (PMC8896342; doi:10.1186/s13023-022-02263-3)
Supplement: Supplementary file 1 — Additional file 1: Table S1. Presents information on the genes analyzed using the Nanostring nCounter platform, including the corresponding reference sequence and target position. [file 13023_2022_2263_MOESM1_ESM.docx]

**Table S1.** Genes included in the panel for analysis by nCounter-Nanostring. The genes associated with the 14 types of MODY described are included, in addition to 5 more related genes (*)

| ***Gene*** | **Description** | **Pathway/Function** | **Phenotype OMIM number** | **RefSeq** | **Target position** |
| --- | --- | --- | --- | --- | --- |
| ***ABCC8*** | ATP binding cassette subfamily C member 8 | Modulator of ATP-sensitive potassium channels and insulin release | 606391 | **NM_001287174.2** | 4856-4955 |
|  |  |  |  | **NM_001351295.2** | 2262-2361 |
| ***APPL1*** | Adaptor Protein, Phosphotyrosine Interacting With PH Domain And Leucine Zipper 1 | Regulation of cell proliferation, and in the crosstalk between the adiponectin signalling and insulin signalling pathways | 616511 | **NM_012096.3** | 751-850 |
| ***BLK*** | Proto-oncogene, Src family tyrosine kinase | Tyrosine-kinase of the src family of proto-oncogenes that has a role in B-cell receptor signaling and B-cell development | 613375 | **NM_001715.3** | 112-211 |
| ***CEL*** | Carboxyl ester lipase | Carboxyl-ester lipase responsible for the hydrolysis of cholesterol esters as well as a variety of other dietary esters | 609812 | **NM_001807.5** | 274-373 |
| ***GCK*** | Glucokinase | Phosphorylation of glucose at the sixth carbon position is the first step in glycolysis. Regulatory role in glucose metabolism | 125851 | **NM_001354800.1** | 22-121 |
|  |  |  |  | **NM_001354801.1** | 5-104 |
|  |  |  |  | **NM_001354802.1** | 153-252 |
|  |  |  |  | **NM_001354803.1** | 33-132 |
|  |  |  |  | **NM_033507.3** | 114-213 |
| ***GLIS3*** | GLIS family zinc finger 3 | Activator and repressor of transcription | 610199 | **NM_001042413.2** | 610-709 |
|  |  |  |  | **NM_152629.3** | 24-123 |
| ***HADH*** | Hydroxyacyl-CoA dehydrogenase | Oxidation of straight-chain 3-HAhydroxyacyl-CoAs as part of the beta-oxidation pathway | 609975 | **NM_001331027** | 1-100 |
|  |  |  |  | **NM_005327.5** | 659-758 |
| ***HNF1A*** | HNF1 homeobox A | Transcription factor required for the expression of several liver-specific genes | 600496 | **NM_000545.6** | 1749-1848 |
|  |  |  |  | **NM_001306179.2** | 2-101 |
| ***HNF1B*** | HNF1 homeobox B |  | 137920 | **NM_00165923.4** | 1677-1776 |
|  |  |  |  | **NM_001304286.2** | 1377-1476 |
| ***HNF4A*** | Hepatocyte nuclear factor 4 alpha |  | 125850 | **NM_001030004.3** | 115-214 |
|  |  |  |  | **NM_178849.3** | 154-253 |
| ***IER3IP1*** | Immediate early response 3 interacting protein 1 | Role in ER stress response by mediating cell differentiation and apoptosis | 614231 | **NM_016097.5** | 141-240 |
| ***INS*** | Insulin | Regulation of carbohydrate and lipid metabolism | 613370 | **NM_000207.3** | 18-117 |
| ***KCNJ11*** | Potassium inwardly rectifying channel subfamily J member 11 | Integral membrane protein and inward-rectifier type potassium channel associated with the sulfonylurea receptor SUR | 616329 | **NM_000525.3** | 348-447 |
|  |  |  |  | **NM_001166290.2** | 44-143 |
| ***KLF11*** | Kruppel like factor 11 | Zinc finger transcription factor that binds to SP1-like sequences in epsilon- and gamma-globin gene promoters that inhibits cell growth and causes apoptosis. | 610508 | **NM_001177716.1** | 1-100 |
|  |  |  |  | **NM_001177718.1** | 44-143 |
|  |  |  |  | **NM_003597.5** | 105-204 |
| ***NEUROD1*** | Neuronal differentiation 1 | NeuroD family of basic helix-loop-helix (bHLH) transcription factors that regulates expression of the insulin gene | 606394 | **NM_002500.4** | 194-293 |
| ***PAX4*** | Paired box 4 | Transcription factor with critical roles during fetal development and cancer growth | 612225 | **NM_001366110.1** | 1223-1322 |
| ***PDX1*** | Pancreatic and duodenal homeobox 1 | Transcriptional activator of several genes, involved in the early development of the pancreas and that plays a major role in glucose-dependent regulation of insulin gene expression | 606392 | **NM_000209.4** | 450-549 |
| ***UCP2*** | Uncoupling protein 2 | Mitochondrial uncoupling protein that separate oxidative phosphorylation from ATP synthesis with energy dissipated as heat, also referred to as the mitochondrial proton leak | 607447 | **NM_003355.2** | 101-200 |
| ***PLAGL1*** | PLAG1 like zinc finger 1 | Zinc finger protein that functions as a suppressor of cell growth | 601410 | **NM_001080951.3** | 54-153 |
|  |  |  |  | **NM_001080952.3** | 265-364 |
|  |  |  |  | **NM_001317156.1** | 1-100 |
|  |  |  |  | **NM_001317157.1** | 42-141 |
|  |  |  |  | **NM_001317159.2** | 101-200 |
|  |  |  |  | **NM_001317162.2** | 187-286 |
|  |  |  |  | **NM_006718.5** | 175-274 |
