# Supplementary material for: Transcriptomic analysis of patients with clinical suspicion of maturity-onset diabetes of the young (MODY) with a negative genetic diagnosis
Source: Orphanet J Rare Dis. 2022 Mar 4;17:105. doi: 10.1186/s13023-022-02263-3 (PMC8896342; doi:10.1186/s13023-022-02263-3)
Supplement: Supplementary file 2 — Additional file 2: Table S2 Presents housekeeping genes included in the panel for nCounter-Nanostring analysis. [file 13023_2022_2263_MOESM2_ESM.docx]

**Table S2.** Housekeeping genes included in the panel for nCounter-Nanostring analysis.

| ***Gene*** | **Description** | **RefSeq** | **Target position** |
| --- | --- | --- | --- |
| ***ABCF1*** | ATP binding cassette subfamily F member 1 | NM_001090.2 | 851-950 |
| ***ALAS1*** | 5'-aminolevulinate synthase 1 | NM_000688.4 | 396-495 |
| ***EEF1G*** | Eukaryotic translation elongation factor 1 gamma | NM_001404.4 | 1151-1250 |
| ***G6PD*** | Glucose-6-phosphate dehydrogenase | NM_000402.2 | 1156-1255 |
| ***GAPDH*** | Glyceraldehyde-3-phosphate dehydrogenase | NM_001256799.1 | 387-486 |
| ***GUSB*** | Glucuronidase beta | NM_000181.3 | 1900-1999 |
| ***HPRT1*** | Hypoxanthine phosphoribosyltransferase 1 | NM_000194.1 | 241-340 |
| ***TBP*** | TATA-box binding protein | NM_001172085.1 | 588-687 |
